# Metabolic patterns predispose human pluripotent stem cells to spatial organization of cell fate

**DOI:** 10.3389/fcell.2025.1696372

**Published:** 2026-01-02

**Authors:** Chunhao Deng, Zhaoying Zhang, Xia Xiao, Carlos Godoy-Parejo, Faxiang Xu, Chengcheng Song, Huanyi Lin, Qinru Li, Shicai Fang, Weiwei Liu, Guokai Chen

**Affiliations:** 1 Centre of Reproduction, Development and Aging, Faculty of Health Sciences, University of Macau, Taipa, Macao SAR, China; 2 Zhuhai UM Science and Technology Research Institute, Zhuhai, Guangdong, China; 3 Institute of Translational Medicine, Faculty of Health Sciences, University of Macau, Taipa, Macao SAR, China; 4 Translational Medicine R&D Center, Biological Imaging and Stem Cell Core Facility, Faculty of Health Sciences, University of Macau, Taipa, Macao SAR, China; 5 MoE Frontiers Science Center for Precision Oncology, University of Macau, Taipa, Macao SAR, China

**Keywords:** tissue pattern, BMP4, cell adhesion matrices, mTOR, metabolic pattern, mitochondrial membrane potential

## Abstract

**Introduction:**

During embryogenesis, specific morphogen gradients are essential for inducing tissue pattern formation. In two-dimensional (2D) human pluripotent stem cell (hPSC) culture, distinct patterns can emerge in hPSC colonies without external morphogen gradients, implying that critical intrinsic factors may induce spatial organization. However, studying the mechanism is challenging due to the lack of efficient spatial labels.

**Methods:**

We employed the mitochondrial membrane potential (MMP) probe JC-1 to stain and track cells within hPSC colonies. Using this tool, we assessed metabolic patterns under different culture coatings and manipulated pathways using mTOR and ROCK inhibitors.

**Results:**

We identified JC-1 as a durable spatial tracker, revealing a clear metabolic pattern in hPSC colonies, significantly influenced by coating materials (integrin-stimulating matrices vs. E-cadherin). This metabolic pattern correlated with spatial mesodermal cell fate under BMP4 induction. Modulation of the mTOR pathway altered the metabolic pattern and subsequent mesoderm induction.

**Conclusion:**

This study reveals that intrinsic metabolic patterns predispose hPSCs to spatial organization of cell fate and highlights JC-1 as a potent spatial marker for studying tissue patterning mechanisms.

## 1 Introduction

Proper tissue pattern formation is essential for successful embryogenesis. In this process, different cell types are induced by specific morphogens (growth factors) and are then spatially organized into distinct patterns. Several factors, including mechanical stress and morphogen gradients, have been studied for their impact on cell fate determination and spatial organization (Winklbauer and Parent, 2017; Etoc et al., 2016; Schwarz and Gardel, 2012; Sevcik et al., 2017); the interplay of these cues with intrinsic cellular states, particularly pre-patterning by metabolism, is less well understood. Due to the complexity of animal models, it is challenging to study pattern formation *in vivo*. Therefore, there is an urgent need for the establishment of simplified *in vitro* models with proper spatial tracking tools that would be helpful for studying this fundamental process.

Human pluripotent stem cells (hPSCs), including human embryonic stem cells (hESCs) and human induced pluripotent stem cells (hiPSCs), resemble postimplant epiblasts and can generate all cell types in the body (Thomson et al., 1998; Xu et al., 2002). hPSCs are indispensable model systems to study key developmental events. hPSCs can be cultured as two-dimensional (2D) aggregates (colonies) on specific extracellular matrices (ECMs) or intercellular adhesion molecules (ICAMs) such as Matrigel or E-cadherin (Liu et al., 2019). hPSC maintenance and differentiation can be modulated by growth factors such as FGF2, TGFβ, and BMP4, which are also common morphogens in embryogenesis (Efthymiou et al., 2014). In 2D culture, all exogenous growth factors are evenly distributed without a gradient. Interestingly, BMP4 can induce a ring-shape pattern of different germ layers in 2D hPSC colonies that are geometrically confined by micropatterns on specific ECM (Etoc et al., 2016; Fan et al., 2016). This observation suggests that 2D hPSC colonies can serve as a good model system to study the mechanism of intrinsic factors that affect pattern formation.

In differentiation, intrinsic heterogeneous factors such as cell metabolism, cell-cycle status, or cell mechanical stress in hPSC colonies can potentially lead to different cell types. Cell metabolism provides the necessary energy and substrates for cell growth and function, thus also leading to cell fate decisions in stem cells (Döhla et al., 2022; Zhang et al., 2018). Metabolic activities and nutrient availability have also been well studied to influence mTOR-mediated signaling, subsequently controlling cell growth, proliferation, and differentiation (Kim and Guan, 2019; Song et al., 2019). Indeed, previous studies have described spatial heterogeneity in metabolic states, such as mitochondrial membrane potential (MMP), within hPSC colonies (Kim et al., 2022; Lau et al., 2020). The cell cycle could also influence cell fate determination by regulating gene expression, signaling pathways, and epigenetic modifications (Madrigal et al., 2023; Soufi and Dalton, 2016). Furthermore, mechanical stress, generated through cell–cell contacts and cell–ECM interactions, varies with a cell’s spatial position and directly impacts fate determination (Muncie et al., 2020). For instance, hPSCs lose epithelial barrier integrity at the edges of colonies, which have been shown to have higher sensitivity to BMP4 (Vasic et al., 2023). In addition, mechanical manipulation of hPSC sub-populations by disruption of cell–cell interactions or cell’s cortical tension results in controllable cell-driven self-organization into repeatable patterns (Libby et al., 2018). On the other hand, the ECM also provides a complex network of proteins and other molecules that surround cells, not only offering structural support but also actively participating in cell signaling (Pompili et al., 2021; Kim et al., 2011; Blin, 2021). Integrin-based focal adhesion could activate the PI3K–AKT pathway (Paul et al., 2020). The adherent junction protein E-cadherin forms a dynamic complex with catenins and regulates several intracellular signal transduction pathways, including Wnt–β-catenin, PI3K–AKT, Rho GTPase, and NF-κB signaling (Van den Bossche et al., 2012).

Previous studies have shown that regulation of cell metabolic flux leads to cell fate decision in hPSCs (Cliff et al., 2017). Studies also revealed that hPSCs can form spatial structures detected through metabolism-associated cell trackers such as MitoTracker or MMP detector after colony formation (Kim et al., 2022; Lau et al., 2020); the connection between these spatial metabolic structures and cell fate during differentiation still requires further investigation. In addition, there is a notable disparity in the mechanical stress experienced by cells in unconfined colonies compared to those in confined colonies. The expansion of hPSC colonies on surfaces coated with ECMs represents a significant contrast to the gastruloid system, in which cells are geometrically confined (Warmflash et al., 2014). The cell adhesion tension in colonies leads to cell fate determination, especially by the shape of the confined structure of a colony (Muncie et al., 2020). It remains unclear whether the mechanism of tissue patterns in confined colonies could be applied to unconfined colonies.

In this study, we aim to identify a fluorescent label to spatially stain and trace hPSCs in colonies. We demonstrate that JC-1 serves as a durable spatial tracker of MMP, enabling us to directly probe the functional role of metabolic patterns. Using this tool, we investigate the molecular regulation of pattern formation and particularly test the connection between a high-MMP state and mesodermal fate bias in both confined and unconfined hPSC colonies.

## 2 Materials and methods

### 2.1 hPSC culture and maintenance

The hPSCs used in this study were the H1 (NIHhESC-10-0043) and H9 (NIHhESC-10-0062) hESC lines, obtained from WiCell Research Institute, Madison, WI, United States. hPSCs were cultured and maintained in E8 medium as previously described (Chen et al., 2011). In brief, H1 cells were cultured in E8 medium on a Matrigel-coated (Corning, NY, United States, 354230) tissue culture-treated plate for maintenance. Cells were passaged with DPBS-EDTA and Y27632 (10 μM).

### 2.2 2D gastrulation on the confined surface

For cell gastrulation on the confined Matrigel surface, Matrigel was diluted with DMEM/F12 (Gibco, Paisley, United Kingdom, 11330-032) by 20-fold and added dropwise (approximately 0.1 μL per drop) to each well of the non-treated plate (Thermo Fisher Scientific, Roskilde, Denmark, 150239). Then the plate was incubated in an incubator 37 °C for 30 min and washed with DMEM/F12 twice. A measure of 500 μL DMEM/F12 was added to the well to prepare it for cell seeding. hESCs were dissociated using TrypLE (Gibco, 12604-021) before they were neutralized with E8 containing 0.5% BSA (Sigma-Aldrich, St. Louis, MO, United States, 7030-100g). hESCs were resuspended in E8 with 10 μM Y27632 (DC Chemicals, Shanghai, China, DC1028); then, approximately 2 × 10^5^ cells/cm^2^ were seeded into every well. Y27632 was removed, and fresh E8 was added 4 h after passaging. Patterned hESCs attached to the Matrigel-coated area within 24 h of seeding.

### 2.3 Mesoderm differentiation

For mesoderm differentiation, hESCs were treated with 20 ng/mL BMP4 (R&D Systems, 314-BP) in E8 medium for 48 h and subsequently harvested for RT-qPCR analysis to detect the expression of the mesoderm markers *TBXT* and *MIXL1*.

### 2.4 RNA extraction and gene expression analysis

Cells were harvested using RNAiso Plus (TAKARA, Dalian, Liaoning, China, 9109) for RNA extraction; RNA (500 ng) was reverse-transcribed with the Maxima H Minus Reverse Transcriptase kit (Applied Biosystems, Foster City, CA, United States, 4368814); cDNA was diluted 20 times in distilled water, and 2 μL was used for gene expression analysis by qPCR using TB Green® Premix Ex Taq™ (TAKARA, RR420) on the ViiA 7 real-time PCR system. Gene expression was normalized to TBP or the control as indicated. The primers used in this study for qPCR are listed in Supplementary Table S1.

### 2.5 Cell staining, FACS analysis, and cell sorting

To study metabolic activities, we included JC-1 [Molecular Probes (Invitrogen), Eugene, OR, United States, T3168], TMRE (Invitrogen, T669), CellROX Green [Invitrogen (Thermo Fisher Scientific), Carlsbad, CA, United States, C10444], MitoTracker Red CMXRos [Invitrogen (Thermo Fisher Scientific), Carlsbad, CA, United States, M46752], and MitoTracker Green FM (Molecular Probes, M7514). The characteristics of different dyes are shown in Table 1. For cell staining, JC-1 (1 μM), TMRE (1 μM), CellROX Green (5 μM), or MitoTracker Red CMXRos (5 μM) was added into cell culture medium and incubated for 30 min, after which the medium was removed and washed with DMEM/F12. Colonies were analyzed 1 h after staining.

**TABLE 1:** Characteristics of different dyes used in this study.

Dye	Function	Staining method	Detection method	Excitation/emission (nm)
JC-1	Mitochondrial membrane potential analysis	Incubation for 30 min and imaging	Live cell imaging/flow cytometry	488/524 (monomer); 540/594 (aggregates)
TMRE	Mitochondrial membrane potential analysis	Incubation for 30 min and imaging	Live cell imaging	540/594
MitoTracker red CMXRos	Mitochondria labeling	Incubation for 30 min and imaging	Live cell imaging	540/594
NBDG	Glucose uptake	Glucose starvation, followed by incubation for 30 min and imaging	Live cell imaging	488/524
CellROX green	Oxidative stress detection	Incubation for 30 min and imaging	Live cell imaging	488/524

For 2-NBDG staining, hESC colonies were cultured in glucose-free medium for 24 h before NBDG staining. NBDG (50 μM) was added into cell culture medium and incubated for 30 min, after which the medium was removed and washed with DMEM/F12. Colonies were analyzed 30 min after staining.

Stained cells were observed in fresh culture medium under an EVOS FL Auto fluorescence microscope before cells were individualized using TrypLE Select for flow cytometry analysis (Beckman Coulter CytoFLEX S flow cytometer) or cell sorting (BD FACSAria™ III cell sorter). Forward scatter (FSC) and side scatter (SSC) parameters were used to exclude debris and aggregates in flow cytometry. Unstained cells were used as a negative control, and four-quadrant gates were used to separate the positively stained cells.

### 2.6 Immunostaining

Cells in the plate were washed using PBS (Gibco, 10010-023), then fixed with 4% PFA (Sigma, 30525-89-4) for 15 min, and finally permeabilized by 0.5% Triton X-100 (Sigma, T8787-100 ML) for 15 min. Antibodies were diluted in PBS with 1% BSA at recommended concentration. Cells were incubated with primary antibodies for 2 h, followed by incubation with secondary antibodies for 1 h at room temperature. Hoechst 33342 was applied for 5 min to stain nuclei before imaging. Antibodies used for TBXT included Brachyury (D2Z3J) rabbit mAb (Cell Signaling Technology, Danvers, MA, United States, 81694).

### 2.7 Western blotting

Cells were lysed in Laemmli buffer (Bio-Rad Laboratories, Hercules, CA, United States, 1610737EDU), and 10 µg total protein was loaded for each sample. After electrophoresis, proteins were transferred onto a PVDF membrane. The membrane was blocked with 5% non-fat milk (Sangon Biotech, Shanghai, China, A600669) in 1× TBST, followed by incubation with primary antibodies overnight at 4 °C and subsequently with secondary antibodies for 1 h at room temperature. Chemiluminescent signals were generated with ECL substrate (Thermo Fisher Scientific, 34580) and detected using a ChemiDoc imaging system (Bio-Rad). Antibodies used in this study included Phospho-mTOR (Ser2448) (D9C2) XP® rabbit mAb (Cell Signaling Technology, 5536) and GAPDH antibody (DSHB-hGAPDH-2G7).

### 2.8 RNA-seq and bioinformatics analysis

Cells were harvested using RNAiso Plus (TAKARA, 9109) for RNA sequencing (RNA-seq) by Azenta. Gene read counts were normalized to transcripts per million (TPM). Log_2_ (TPM of each gene in each sample/mean TPM of each gene in all samples) values were used to generate heatmaps using the R package gplots. Differentially expressed genes (DEGs) were selected using the R package edgeR based on a p-value <0.01 and fold change >1.5 or <−1.5. The heatmap cluster was established using the Euclidean distances between selected genes in each sample. Gene set enrichment analysis was carried out using Enrichr (https://maayanlab.cloud/Enrichr/) (Kanehisa et al., 2023; Chen et al., 2013; Kuleshov et al., 2016).

### 2.9 Statistical analysis

Data are presented as mean ± SD of three or more biological replicates. Statistical analysis was carried out using one-way ANOVA in Prism. P < 0.05 was considered statistically significant.

## 3 Results

### 3.1 Identification of a metabolic dye that enables spatial labeling and tracking of cells in hPSC colonies

**FIGURE 1:**
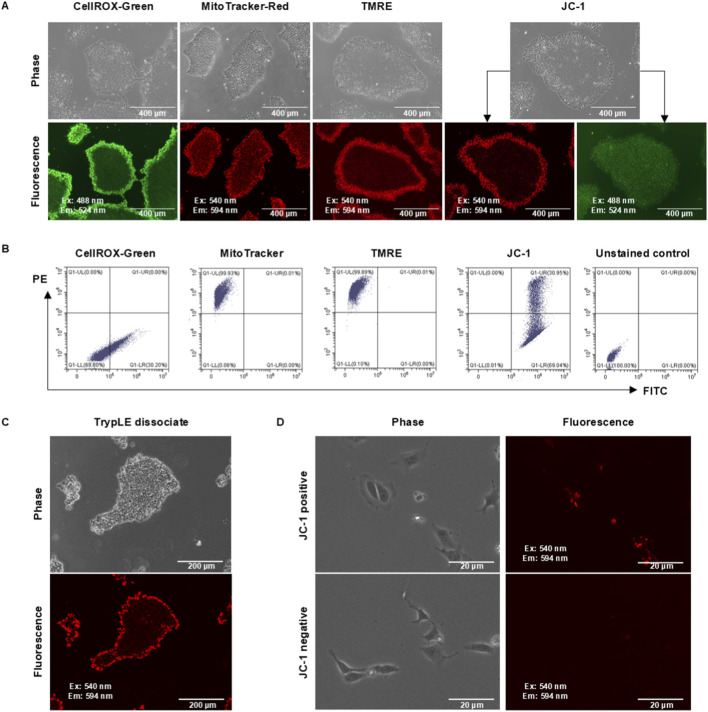
Identification of a fluorescent probe for spatial tracing. **(A)** Staining patterns using different fluorescent probes. H1 hPSCs were cultured in E8 medium on the Matrigel-coated surface for 3 days before they were stained and observed under a fluorescence microscope. **(B)** The stained cells were analyzed by flow cytometry. **(C)** Staining patterns during individualization. After hPSC colonies were stained using JC-1 dye, they were dissociated using TrypLE and observed under a microscope before cell collection. **(D)** JC-1 stain after cell sorting. After hPSC colonies were stained with JC-1, cells were individualized with TryPLE, sorted by flow cytometry, and the two populations seeded back onto the Matrigel-coated surface. Cell morphology and fluorescent signals were observed 6 hours after plating.

To study pattern formation in hPSCs, we first used H1 hESCs to identify dyes that could spatially track cells in hPSC colonies. Three days after hESC colonies were formed on the Matrigel-coated surface, various fluorescent dyes were applied to cells for observation through fluorescence microscopy. Multiple metabolism-associated dyes were found to spatially label hPSC colonies (Figure 1A; Supplementary Figure S1D). The outer rings of the colonies were specifically stained using CellROX Green and MitoTracker Red, along with mitochondrial membrane potential dyes TMRE (tetramethylrhodamine, ethyl ester) and JC-1 (5,5ʹ,6,6ʹ-tetrachloro-1,1ʹ,3,3ʹ-tetraethylbenzimidazolylcarbocyanine iodide) (Figure 1A). JC-1 staining showed that green JC-1 monomers (emission wavelength 524 nm) were evenly distributed in the whole colony, but red JC-1 aggregates (J-aggregates, emission wavelength ∼594 nm) specifically stained the outer ring (Figure 1A; Supplementary Figure S1C), which is an indicator of elevated MMP. For simplicity, we refer to cells with red JC-1 aggregates as JC-1-positive cells in this study. Different from mitochondrial dyes, the glucose uptake indicator 2-NBDG (2-deoxy-2-[(7-nitro-2,1,3-benzoxadiazol-4-yl)amino]-D-glucose) exhibited an even distribution throughout the colony, indicating that this phenotype is specific to mitochondrial dyes (Supplementary Figure S1A). At the same time, results with FUCCI cell-cycle reporter demonstrated that green S/G_2_/M-phase cells and red G_1_-phase cells did not form a pattern resembling JC-1 staining (Supplementary Figure S1B). These data suggested that hPSC colonies were spatially patterned with elevated MMP in outer ring cells, but cell-cycle status and glucose uptake were uniform across the entire colony, regardless of the location of the cells in the colony.

To test the feasibility of using these dyes for cell tracking, we investigated whether cells could maintain the fluorescent signals during cell culture manipulations. After colonies were stained with the indicated dyes, the cells were dissociated and analyzed through flow cytometry (Figure 1B). Uniform populations were observed in cells stained with CellROX, MitoTracker, and TMRE. It implied that those stained cells lost their original spatial label after dissociation, and these dyes were not suitable spatial trackers for pattern-formation studies. In contrast, two distinct cell populations were detected by flow cytometry in JC-1-stained cells, indicating that the fluorescence signal was retained after cell dissociation. Further observations showed that under enzyme/EDTA dissociation, colonies disintegrated, but stained cells in the outer ring maintained the red fluorescence (Figure 1C). Even after cell sorting and replating, the red fluorescence was maintained in JC-1-positive cells, while negative cells did not gain additional red fluorescence after dissociation (Figure 1D). These data suggested that JC-1-stained cells maintained the spatial information at the time of staining and could be used for spatial tracking of hPSCs.

### 3.2 Spatial organization of JC-1-positive cells in cell culture

**FIGURE 2:**
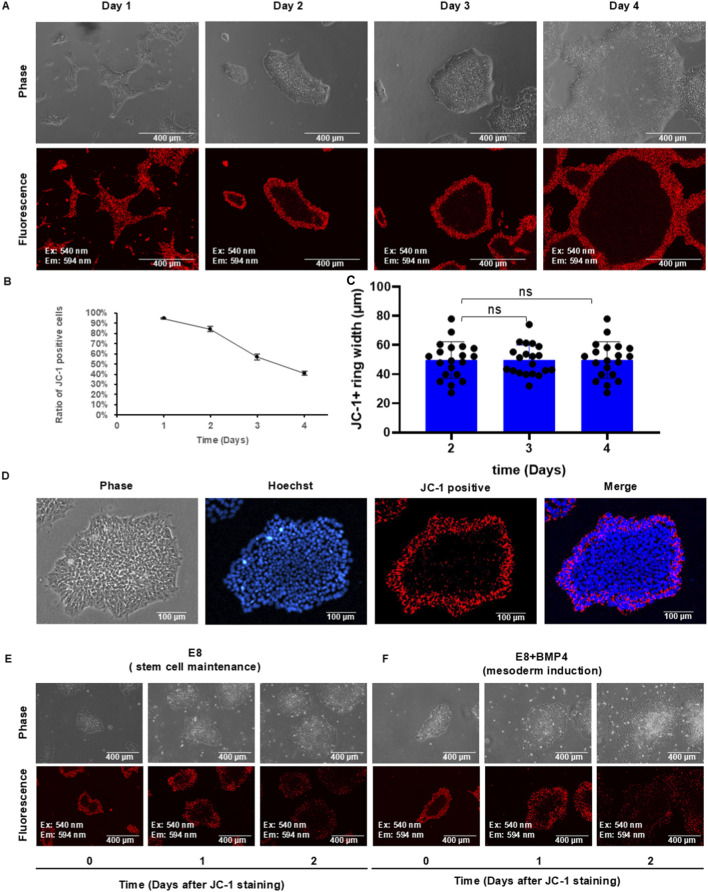
Spatial patterns of hPSCs on the Matrigel-coated surface during maintenance and differentiation. **(A–C)** Time course of JC-1 pattern changes during maintenance. hPSCs were passaged onto the Matrigel-coated surface using DPBS-EDTA, and they were stained with JC-1 daily before analysis by microscopy **(A)** and flow cytometry **(B)**. Flow cytometry data are presented as mean ± SD of three biological replicates **(C)**. ImageJ was used to measure the width of the JC-1-positive ring in hPSC colonies. n = 20 data points on the image. **(D)** Comparison of nuclear staining (Hoechst) and JC-1 staining in hPSC colonies to show the width of the ring. **(E)** JC-1-based cell tracing during maintenance. hPSC colonies were stained with JC-1 briefly and were cultured and observed in E8 medium. **(F)** JC-1-based cell tracing during BMP4-induced differentiation. hPSC colonies were stained with JC-1 briefly and were cultured and observed in E8 medium with BMP4 (20 ng/mL).

We utilized JC-1 to observe tissue patterning of hPSCs on the Matrigel surface during maintenance in E8 medium. Preliminary experiments showed that once JC-1 dye is washed away, the cellular signal decreases over time and becomes undetectable after 3 days in culture (Supplementary Figure S2C); for this reason, the cells were stained daily (Figure 2A). One day after passaging, small colonies formed, and almost all cells were JC-1-positive (Figure 2A). In the following days, a JC-1-negative zone appeared in the center of each colony. The apparent decrease in the proportion of positive cells (Figure 2B) is not due to loss of the dye signal, but rather to the colonies increasing in size. Moreover, the dye labels the cells located at a fixed distance from the colony border, regardless of colony size (Figure 2C). Given that the circumference increases linearly with colony diameter whereas the area grows exponentially with colony diameter, the ratio of labeled cells decreases as the colonies increase in size. Compared with nuclear staining, the outer ring of the colony remained two cells thick during colony expansion (Figure 2D; Supplementary Figure S2A). This observation is consistent with the theoretical prediction of a two-cell-thick outer ring (Supplementary Figure S2B). Taken together, these data suggested that hESCs are spatially organized within colonies and cells in the two-cell-thick outer layer have higher MMP than those inside the colony.

To analyze cell movement in colonies, we first assessed the dynamics of JC-1-positive cells during cell culture. After a brief exposure to JC-1, cells were maintained in E8 medium and observed daily. The results showed that JC-1-positive cells could be easily visualized 2 days after staining (Supplementary Figures S2D–F). Furthermore, although JC-1 staining exhibited minimal toxicity during BMP4-induced differentiation, the differentiation outcomes were similar to those of unstained control cells (Supplementary Figures S2H,I). Having established these parameters, we then used JC-1 to track hPSCs during regular maintenance and differentiation (Figures 2E,F). As colonies grew larger, the JC-1-positive ring expanded and remained at the colony’s periphery. In mesoderm differentiation under BMP4 induction, JC-1-positive cells also remained in the outer ring during differentiation (Figure 2F). These data suggested that cells in hPSC colonies maintained their relative position during maintenance and differentiation.

### 3.3 Transcriptome analysis of spatial gene expression in colonies during maintenance and differentiation

**FIGURE 3:**
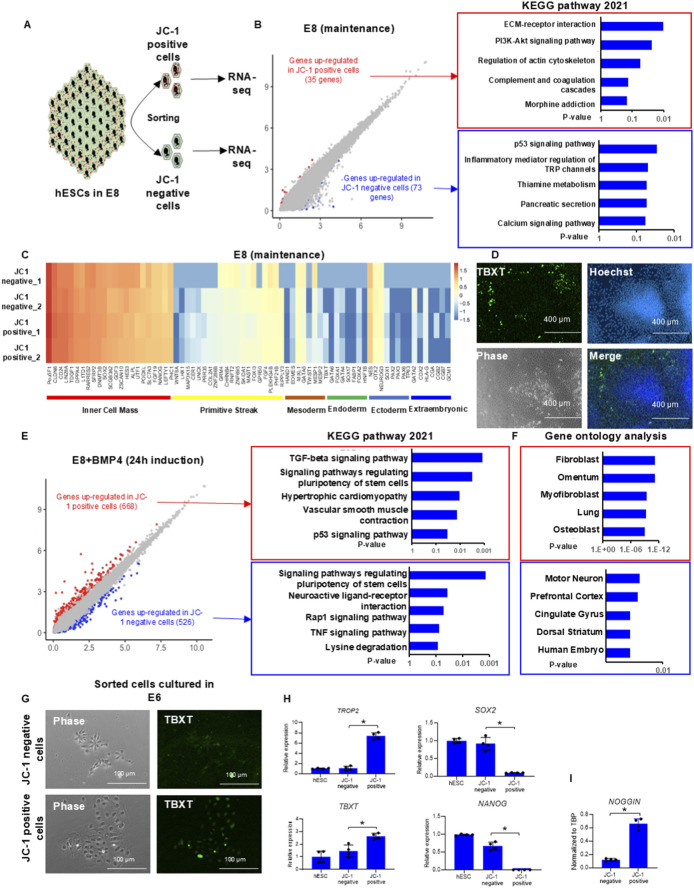
Transcriptome analysis of spatial gene expression in hPSC colonies. **(A)** Schematic illustration of RNA-seq analysis after JC-1-based cell sorting. **(B)** Volcano plot of differentially expressed genes and the enriched KEGG pathways between JC-1-positive and JC-1-negative cells from hPSC colonies in E8 medium (Kanehisa et al., 2023), analyzed using Enrichr. **(C)** Lineage-specific gene expression between JC-1-positive and JC-1-negative cells from hPSC colonies. **(D)** Mesoderm marker TBXT (Brachyury) expression in hPSC colony after BMP4 induction. hPSC colonies were differentiated under 20 ng/mL BMP4 in E8 for 48 h and were immunostained with the anti-TBXT antibody. **(E)** Volcano plot of differentially expressed genes and the enriched KEGG pathways between JC-1-positive and JC-1-negative cells from hPSC colonies 24 h after BMP4 (20 ng/mL) induction in E8 medium (Kanehisa et al., 2023), analyzed using Enrichr. **(F)** Gene Ontology analysis of differentially expressed genes between JC-1-positive and JC-1- negative cells 24 h after BMP4 (20 ng/mL) induction. **(G,H)** Cell morphology and gene expression of sorted cells from **(F)** after spontaneous differentiation. **(G)** Sorted JC-1-positive and JC-1-negative cells were cultured in E6 for 2 days, and RNAs were collected for analysis through RT-qPCR. **(H)** qPCR analysis of the expression of lineage markers *TROP2*, *TBXT*, *SOX2*, and *NANOG* in sorted cells. Data are presented as mean ± SD of four independent experiments. **(I)** qPCR analysis of *NOGGIN* expression in sorted JC-1-positive cells. Data are presented as mean ± SD of four independent experiments.

Next, we investigated the global gene expression in cells from different locations in a colony. hESC colonies were dissociated, and JC-1-positive (outer ring) and JC-1-negative (center) populations were sorted out for RNA-seq analysis (Figure 3A). More than 100 genes were differentially expressed between the two populations. A total of 35 genes were elevated in JC-1-positive cells, and KEGG analysis showed their enrichment in ECM–receptor interaction, PI3K–AKT signaling pathway, and regulation of actin cytoskeleton. A total of 73 genes were upregulated in JC-1-negative cells, which were associated with p53 signaling pathway, inflammatory mediator, regulation of TRP channels, thiamine metabolism, and calcium signaling pathway (Figure 3B). The expression levels of most pluripotency and lineage markers were comparable between the two populations (Figure 3C). These data suggested that hESC pluripotency was not dependent on the spatial location in a colony.

We then inspected pattern formation in hPSC colonies on the regular Matrigel-coated surface where cells can migrate more freely than in gastruloids. Similar to BMP4-treated gastruloids (Etoc et al., 2016; Warmflash et al., 2014), the mesoderm marker TBXT (Brachyury, T) was detected in the outer ring of each colony 2 days after the induction (Figure 3D). The location of TBXT-positive cells in colonies is similar to that of JC-1-positive cells before induction, which are located in the outer ring (Figure 2F). This suggested that the patterns of mesoderm induction correlated with the metabolic pattern at the beginning of the differentiation.

To understand the differential induction pattern, we investigated spatial gene expression before the lineage pattern was finally determined. Since BMP4 did not alter mitochondrial mass during differentiation (Supplementary Figures S3A,B), we performed cell sorting after BMP4 treatment. One day after BMP4 induction, JC-1-positive and -negative cells were sorted and analyzed through RNA-seq (Figure 3E). Compared to the maintenance condition (E8), significantly more genes were differentially expressed between JC-1-positive and JC-1-negative populations after BMP4 treatment. A total of 668 genes were elevated in JC-1-positive cells, enriched in functions such as TGFβ signaling pathway, regulation of pluripotency of stem cells, hypertrophic cardiomyopathy, vascular smooth muscle contraction, and p53 signaling pathway. A total of 526 genes were upregulated in JC-1-negative cells, and they were enriched in signaling pathways regulating pluripotency of stem cells, neuroactive ligand–receptor interaction, Rap1 signaling pathway, TNF signaling pathway, and lysine degradation (Figure 3E). JC-1-positive cells had elevated gene expression in mesoderm cell types, while JC-1-negative cells had gene expression enriched in human embryo and ectoderm lineage (Figure 3F). The sorted JC-1-positive and -negative cells were replated onto the Matrigel surface in E6 medium (E8 medium without FGF2 and TGFβ1) for spontaneous differentiation. After 48 h, JC-1-positive cells from the outer ring displayed an enlarged cell body (Figure 3G, phase-contrast image) and expressed higher levels of TBXT (Figure 3G, fluorescent image). qPCR analysis showed higher expression of differentiation markers such as *TBXT* and *TROP2* and lower expression of pluripotency markers such as *NANOG* and *SOX2* in JC-1-positive cells (Figure 3H), suggesting that this population is more prone to differentiation. Interestingly, significantly higher *NOGGIN* was expressed in the JC-1-positive population (Figure 3I). In comparison, JC-1-negative cells from the center of colonies had typical hPSC morphology without TBXT expression. This observation is consistent with the results of transcriptome analysis that JC-1-positive cells tend to become committed to mesoderm fate, while JC-1-negative cells were less prone to differentiation. These data suggested that hPSCs in the outer ring of the colonies with higher MMP were more sensitive to BMP4 induction toward mesoderm fate, while cells located in the center differentiated slowly; this observation aligns with studies previously reported.

### 3.4 Cell adhesion-promoting factors affect metabolic and cell fate patterns

**FIGURE 4:**
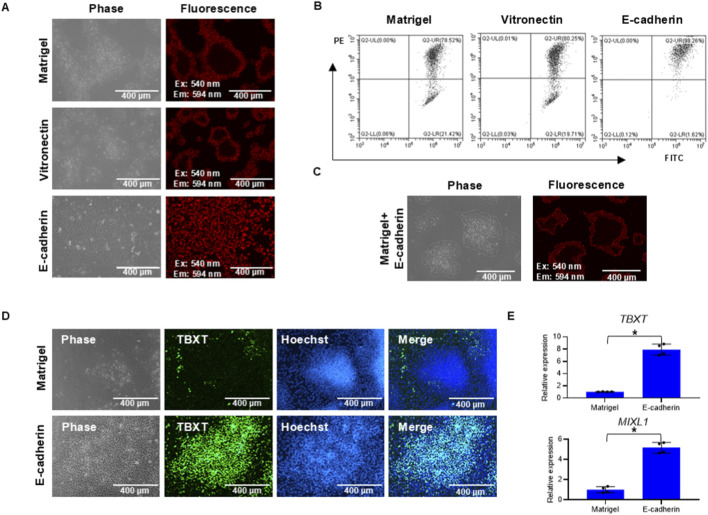
Cell adhesion-promoting factors affect metabolic and cell fate patterns. **(A,B)** Effect of the coating surface on the JC-1 pattern in hPSC colonies. hPSCs were plated onto a Matrigel- or vitronectin- or E-cadherin-coated surface and cultured for 2 days, before they were stained with JC-1 and analyzed using fluorescence microscopy **(A)** and flow cytometry **(B,C)**. H1 hPSC colonies were cultured on the surface containing both Matrigel and E-cadherin for 2 days and stained with JC-1. **(D,E)** Cell adhesion-promoting factors affected mesoderm differentiation induced by BMP4. H1 hPSCs were cultured for 2 days on surfaces coated with either Matrigel or E-cadherin, prior to a 2-day induction with BMP4. Differentiation toward mesoderm was assessed by immunostaining for the marker TBXT **(D)**. Mesoderm markers TBXT (T) and MIXL1 were analyzed using RT-qPCR **(E)** (n = 4, *P < 0.05).

Our data reveal that cells at the colony edge and center exhibit distinct signatures in ECM–receptor interaction, PI3K–AKT signaling, and actin cytoskeleton remodeling (Figure 3B). We propose that these differences reflect a mechanical disparity, which, through mechanotransduction, predisposes the central cells to a specific metabolic state that inhibits mesoderm commitment and favors ectodermal enrichment, thereby explaining the spatial pattern of TBXT emergence. As the above phenotypes were observed on Matrigel, we investigated whether a colony would be organized in a similar way on other surfaces. In contrast to the colony on Matrigel, JC-1-positive cells did not form a ring pattern on E-cadherin surfaces. Instead, JC-1 staining was uniformly distributed throughout the colony in both H1 and H9 cells (Figure 4A; Supplementary Figure S4C). In comparison, the ring pattern was observed on Matrigel- and vitronectin-coated surfaces, both of which act through the integrin pathway (Figure 4A). Flow cytometry analysis showed that a higher percentage of cells on E-cadherin were JC-1-positive than those on Matrigel and vitronectin surfaces (Figure 4B). These data suggested that the metabolic pattern is differentially affected by adhesion matrices. When Matrigel or vitronectin content was decreased, more JC-1-positive cells appeared. When Matrigel was added onto the E-Cadherin surface, the ring-shaped pattern appeared again (Figure 4C), and two distinct populations reappeared in flow cytometry analysis (Supplementary Figure S4A). These data suggested that integrin activation could help epithelial colonies establish the tight junction, which in turn suppresses MMP in the center of colonies to generate the spatially organized metabolic pattern.

We further examined the impact of extracellular matrices on cell fate determination. Under BMP4 induction, expression of early mesoderm markers *TBXT* and *MIXL1* was significantly higher on E-cadherin than on Matrigel surfaces (Figures 4D,E and Supplementary Figure S3B). On Matrigel-coated surfaces, TBXT was expressed in the outer ring of the colony but not in the center. In contrast, TBXT-positive cells were observed throughout the whole colony on E-cadherin (Figure 4D; Supplementary Figure S4D). Additionally, the expression levels of the mesoderm markers TBXT and MIXL1 were significantly higher on E-cadherin (Figure 4). These data indicate that extracellular matrices had a significant impact on metabolic patterning, which in turn affected the pattern of cell fate.

### 3.5 mTOR and ROCK modulate metabolic pattern formation in hPSC colonies

**FIGURE 5:**
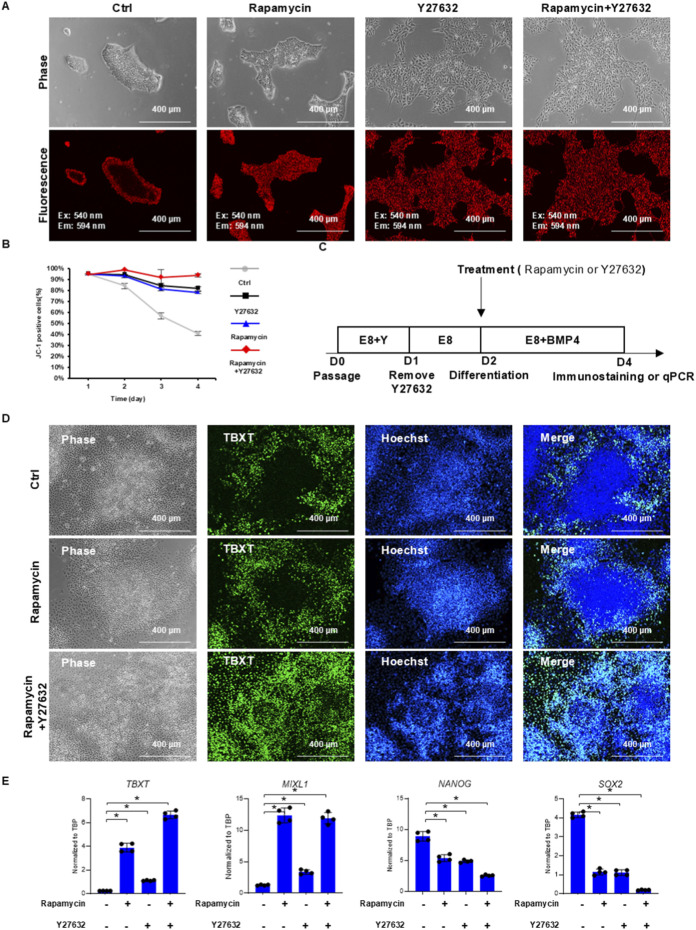
mTOR and ROCK modulate metabolic and cell fate pattern formation in hPSC colonies. **(A,B)** JC-1 patterns under mTOR and ROCK inhibition. hPSCs were passaged with 10 µM Y27632 onto Matrigel-coated surfaces in E8 medium. Cells were then treated with either 100 nM rapamycin (mTOR inhibitor) or 10 µM Y27632 (ROCK inhibitor) on day 1 and day 2. JC-1 staining and microscopy observation were performed on day 3. Cells were also stained with JC-1 and analyzed by flow cytometry daily to monitor mitochondrial membrane potential changes **(B)**. **(C,D)** TBXT immunostaining in BMP4-induced H1 hPSCs. Immunostaining of TBXT on day 4, following BMP4 induction on day 2, with or without rapamycin or Y27632 treatment. **(E)** RT-qPCR analysis of mesoderm induction under different treatments on day 4 of differentiation following BMP4 induction on day 2 (n = 4, *P < 0.05).

Because of the distinct patterns on integrin-stimulating surfaces (Matrigel and vitronectin), we utilized JC-1 to study pattern formation on Matrigel under signaling modulation. Since mTOR inhibition promotes mesoderm differentiation (Song et al., 2019) (Supplementary Figure S5A), mTOR signaling was well known to have a strong relationship with metabolism and has been reported to activate MMP (Fan et al., 2016; Sempou et al., 2022). We also examined whether mTOR inhibitor rapamycin affects metabolic patterning. Rapamycin treatment significantly widened the JC-1 outer ring, and the size of the central zone was decreased (Figure 5A). In addition, because the ROCK pathway is important to colony integrity, we then inspected whether ROCK inhibitor Y27632 could also affect the JC-1 staining pattern. Y27632 treatment resulted in uniform staining of the whole colony (Figure 5A), indicating that MMP was also regulated by the cell–cell interaction. These data suggested that mTOR and ROCK pathways were important for the formation of metabolic patterns in a colony.

A time-course experiment was conducted to further analyze the impact of mTOR and ROCK inhibition during hPSC expansion. After hPSCs were passaged on the Matrigel surface, colonies were treated by a brief exposure to JC-1. Cells were maintained in E8 medium and harvested for analysis every day. In control experiments, the percentage of JC-1-positive cells decreased rapidly every day (Figure 5B), and positive cells were located in the outer ring of colonies (Figure 5A). Rapamycin and Y27632 each elevated the percentage of JC-1-positive cells (Figure 5B) and enlarged the JC-1-positive zone (Figure 5A). When both treatments were applied together, they synergistically improved JC-1 staining (Figures 5A,B). Interestingly, we noticed that ROCK or mTOR inhibition could not reverse the JC-1-stained pattern if the pattern had been formed previously (Supplementary Figures S5B–E). These data suggested that ROCK and mTOR pathways were essential for the initiation of metabolic patterning associated with JC-1 but not for the maintenance of the pattern.

### 3.6 mTOR and ROCK control differentiation pattern formation in hPSC colonies

We then examined the cell fate induction pattern under rapamycin treatment at different stages of metabolic pattern formation. If BMP4 was applied on day 2 after the outer ring formed (Figure 2A, Day 2), TBXT-positive cells emerged only from cells in the outer ring area with or without rapamycin or Y27632 treatment, and a TBXT-negative central zone was clearly visible (Figures 5C,D; Supplementary Figure S5D). This pattern can be altered by the application of both rapamycin and ROCK inhibitor from day 1 prior to BMP4 induction, and TBXT-positive cells became evenly distributed in the colony (Figure 5D, rapamycin + Y27632 and Supplementary Figure S5F). ROCK or mTOR inhibition reversed the differentiation pattern, consistent with the results of gene expression analysis. qPCR analysis demonstrated enhanced differentiation by rapamycin and ROCK inhibitor treatment (Figure 5E). These data suggested that ROCK and mTOR inhibition affected the differentiation pattern in a way similar to their influence on JC-1 metabolic patterning.

### 3.7 Distinct regulation of metabolism and differentiation patterns in confined colonies

**FIGURE 6:**
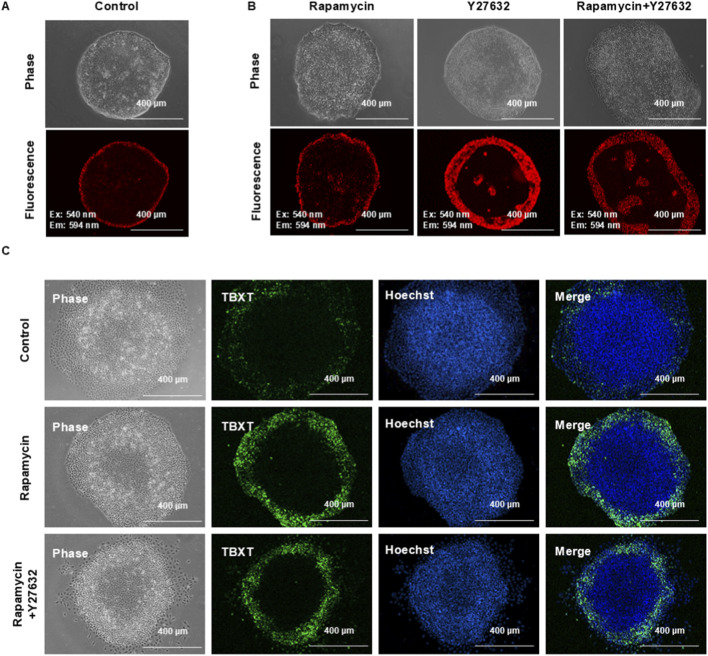
Distinct pattern regulation of metabolism and differentiation in confined colonies. **(A,B)** hESCs were seeded on the confined, Matrigel-coated surface in E8 on day 0 **(A)**. Cells were then treated with either 100 nM rapamycin (mTOR inhibitor) or 10 µM Y27632 (ROCK inhibitor) on day 1, and the JC-1 pattern was recorded on day 2. **(C)** TBXT pattern induced by BMP4 on the confined surface. hESCs were seeded on the confined, Matrigel-coated surface on day 0 and were differentiated with 20 ng/mL BMP4 on day 1 with either 100 nM rapamycin (mTOR inhibitor) or 10 µM Y27632 (ROCK inhibitor). TBXT expression was analyzed by immunostaining on day 3.

The gastruloid model is widely used to study hPSC pattern formation. In this system, colonies are formed on the confined surface where cells do not migrate freely, and cells may display different behavior. We used JC-1 to examine pattern formation in the gastruloid platform for comparison with regular hESC colonies. We showed that JC-1-positive cells were localized in the outer ring (Figure 6A), and the width was similar to colonies on the unconfined surface (Supplementary Figure S6A). Despite enhancing the JC-1-positive ring in confined colonies (Figure 6B; Supplementary Figure S6B), ROCK and mTOR inhibitors failed to recapitulate the JC-1 pattern observed in unconfined colonies (Figure 6B versus 5A). In addition, these inhibitors could not change the TBXT expression pattern under BMP4 induction (Figure 6C versus 5D). These data suggested that hPSCs in confined and unconfined colonies were under different regulation in pattern formation.

In short, we reveal a metabolic pattern of mitochondrial activities in hPSC colonies. Our study suggests that the metabolic pattern predisposes hPSCs to spatial organization of cell fate under BMP4 induction.

## 4 Discussion

Proper tissue patterning is essential in embryogenesis, and its formation often requires growth factor gradients and cell type-specific cell sorting. In this study, we demonstrate a metabolic pattern with MMP heterogeneity in hPSC colonies, which correlates with the cell fate propensity under specific induction. The metabolic status of hPSCs in a colony precludes the emergence of the spatial pattern of the mesoderm cell type.

Mitochondrial activities are associated with ATP production, cell apoptosis, and reactive oxygen species (ROS) generation. Our results demonstrate that cells on the edge of the colony have higher MMP and higher ROS production activity (Figure 1A), which is consistent with the findings in a few recent studies (Kim et al., 2022; Lau et al., 2020; Bothun and Woods, 2020). Building upon recent advances linking mitochondrial dynamics to lineage specification (Döhla et al., 2022; Zhang et al., 2018; Mandal et al., 2011), we further demonstrate JC-1’s utility as a spatial reporter for metabolically primed cell states. Our sequencing data revealed distinct patterns of ECM–receptor interaction, PI3K–AKT signaling, and actin cytoskeleton organization between the colony periphery and center, suggesting that the extracellular microenvironment may influence cell fate through its effects on mechanical properties. We also show that hPSC colonies display distinct metabolic patterns on different cell adhesion-promoting factor-coated surfaces. A heterogeneous MMP pattern with a ring structure is observed only on Matrigel and vitronectin surfaces that activate integrins but not on the E-cadherin surface (Figure 4A). Since previous studies have shown that CDH1 knockout alters the cell distribution pattern in colonies (Libby et al., 2018), it seems that cell adhesion-promoting factors could change the mechanical properties of cells that further affect cell-fate determination.

The 2D gastruloid model (Etoc et al., 2016; Warmflash et al., 2014; Phan-Everson et al., 2021) and this study show that intrinsic factors can play critical roles in pattern formation besides conventional growth factor gradients. Our study reported that cells with higher MMP on the edge are more sensitive to BMP4 induction of mesodermal fate, while cells in the center specify to other cell fate. We show that the mesoderm zone in the outer ring is induced on-site without cell sorting. Our results further demonstrate that combined mTOR and ROCK inhibition alters both metabolic patterning and differentiation outcomes. Mechanistically, the mTOR pathway has been implicated in both metabolic regulation and mechanical property modulation (Paglin et al., 2005; Hornberger, 2011). Similarly, ROCK inhibition perturbs cellular mechanical properties (Libby et al., 2018). It is possible that integrin influences the formation of cell metabolic and fate patterns through mTOR pathways by providing heterogeneous mechanical properties within cell colonies. More work is necessary to learn the molecular mechanism in the formation of metabolic patterns and how it influences cell fate patterns upon differentiation induction.

A confined gastruloid model with hPSCs is widely used to understand signaling regulation in human embryogenesis (Deglincerti et al., 2016; Warmflash et al., 2014; Siggia and Warmflash, 2018). It is important to consider whether the confined gastruloid model could closely reflect the biological processes in such context. Compared to unconfined colonies, the confined gastruloid platform establishes a geometrically defined microenvironment for hPSC growth and differentiation, exhibiting distinct biomechanical properties. Our findings reveal divergent responses to mTOR and ROCK inhibition in metabolic patterning and differentiation outcomes between confined and unconfined colonies (Figures 5, 6). Given the stark differences in mechanical properties, the application of the *in vitro* gastruloid model requires careful consideration regarding its physiological relevance. This comparative analysis underscores the necessity of accounting for mechanical context when modeling embryogenic processes, suggesting that confined gastruloids may not be less relevant but rather represent a distinct mechanical niche.

This study also highlights JC-1 as a unique tracking marker for spatial information in cell culture. JC-1 aggregates imply elevated MMP in cells located in the outer rings of stem cell colonies. However, other trackers quickly lose their original signal and spatial information after digestion or other mechanical manipulations (Figure 1B), but JC-1 aggregates maintain their intensity continuously, even after dissociation. This unique feature of JC-1 allows us to spatially label cells in the outer ring and study their behaviors during maintenance, passaging, and differentiation. Although it is a useful spatial tracker, JC-1 has its limitations. First, JC-1 generally stains two layers of cells in the outer ring, making it unsuitable for specifically studying the cells on the edge of a colony. Second, the JC-1 signal fades away after a couple of days, which prevents its use for *in vivo* studies. For long-term studies *in vitro* or *in vivo* using JC-1 staining, cells from different populations should be sorted out by flow cytometry. Thus, it would be beneficial to identify more specific spatial trackers with better durability in the near future.

In summary, our work demonstrates that a spatial MMP gradient precludes the pattern formation of fate decisions in hPSC colonies. It provides a new perspective on the relationship between metabolic regulation and cell fate determination.

## Data Availability

The datasets presented in this study can be found in online repositories. The names of the repository/repositories and accession number(s) can be found below: NCBI BioProject: https://www.ncbi.nlm.nih.gov/bioproject/PRJNA1070787.
