# Supplementary material for: Metabolic patterns predispose human pluripotent stem cells to spatial organization of cell fate
Source: Front Cell Dev Biol. 2026 Jan 2;13:1696372. doi: 10.3389/fcell.2025.1696372 (PMC12808486; doi:10.3389/fcell.2025.1696372)
Supplement: Supplementary file 1 [file DataSheet1.pdf]

# Metabolic patterns predispose human pluripotent stem cells to spatial organization of cell fate

**Chunhao Deng[1,2,#], Zhaoying Zhang[1,#], Xia Xiao[1], Carlos Godoy-Parejo[1], Faxiang Xu[1], Chengcheng Song[1], Huanyi Lin[2], Qinru Li[2], Shicai Fang[1,2], Weiwei Liu[1,3,4], Guokai Chen[1,2,3,5,*]**

1 Centre of Reproduction, Development and Aging, Faculty of Health Sciences, University of Macau, Macau SAR, China

2 Zhuhai UM Science & Technology Research Institute, Zhuhai, Guangdong, China.

3 Institute of Translational Medicine, Faculty of Health Sciences, University of Macau, Macau SAR, China

4 Biological Imaging and Stem Cell Core Facility, Faculty of Health Sciences, University of Macau, Macau SAR, China

5 MoE Frontiers Science Center for Precision Oncology, University of Macau, Macau SAR, China

# These authors contributed equally to this study

*** Correspondence:**

Corresponding Author

guokaichen@um.edu.mo

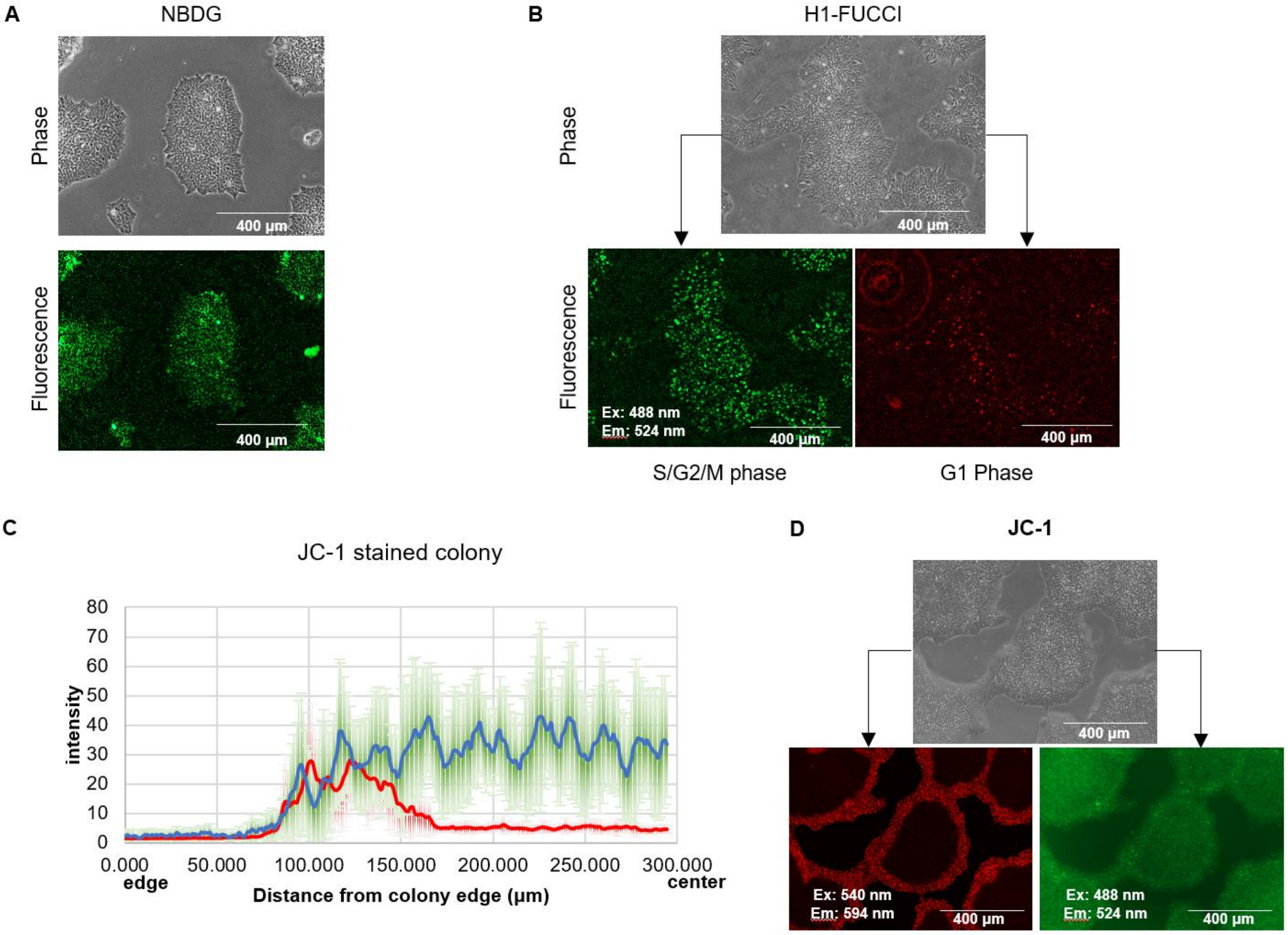


**Supplementary Figure 1.** A. NBDG staining patterning showed no obvious pattern ring in hESC colonies. hPSCs were cultured in E8 medium on Matrigel-coated surface for 3 days before they were stained by NBDG and observed under fluorescence microscope.

B. S/G2/M-phase cells and G1-phase cells were evenly distributed in the PSC colony. H1-FUCCI cells were cultured in E8 medium on Matrigel coated surface for 3 days and were observed under fluorescence microscope.

C. Line-scan profile of JC-1 stained colony analysis by imageJ.

D. JC-1 staining patterns in hESC H9 cells. H9 cells were cultured in E8 medium on Matrigel-coated surfaces for 3 days prior to JC-1 staining and fluorescence microscopy imaging.

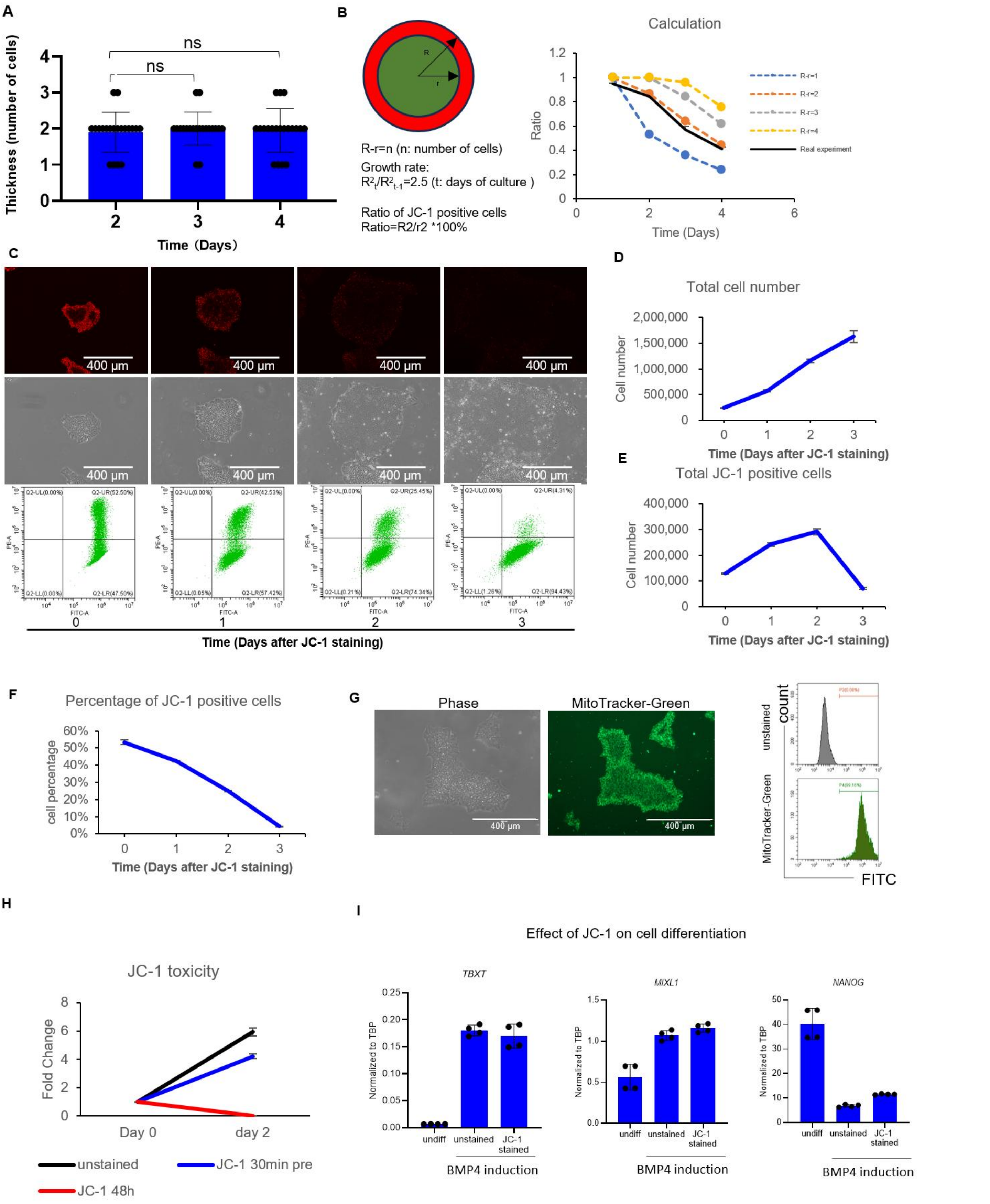


**Supplementary Figure 2.**

A. Number of JC-1 positive cells in the outer ring counted from JC-1 staining at different

days. The number of JC-1 positive cells were counted in hPSC colonies. n = 20 data points on the image.

B. Modeling of JC-1 ratio dynamics during hPSC expansion. Using a theoretical model with a daily growth rate of 2.5 and an initial layer number of 1, we calculated the daily JC-1 ratio for scenarios where the cell layer number (n) reached 1, 2, 3, and 4. The experimental data were best approximated by the model when n=2.

C-F. The JC-1 signal attenuated after the dye was washed away. hESC colonies were stained once with JC-1 after colony formation, and the staining pattern was observed using a fluorescence microscope (scale bar = 400 μm) and FACS (n = 3) every day (B). The total cell count (C), total JC-1 positive cells (D), and percentage of JC-1 positive cells (E) were analyzed based on the FACS data (n=3).

G. MitoTracker-Green staining in hPSCs. hPSC H1 cells were cultured in E8 medium on Matrigel-coated surfaces until colony formation. The MitoTracker Green staining pattern was observed by fluorescence microscopy and quantified by flow cytometry (FACS).

H-I. Effect of JC-1 staining on hPSC growth and differentiation during BMP4-induced differentiation. hPSC colonies were briefly stained with JC-1 and then cultured in E8 medium supplemented with 20 ng/mL BMP4. Cell numbers were monitored to calculate the growth fold change (H), BMP4 responsive lineage specification was evaluated via qPCR for *NANOG* (pluripotency) and *TBXT/MIXL1* (differentiation) markers (I) (n=4).

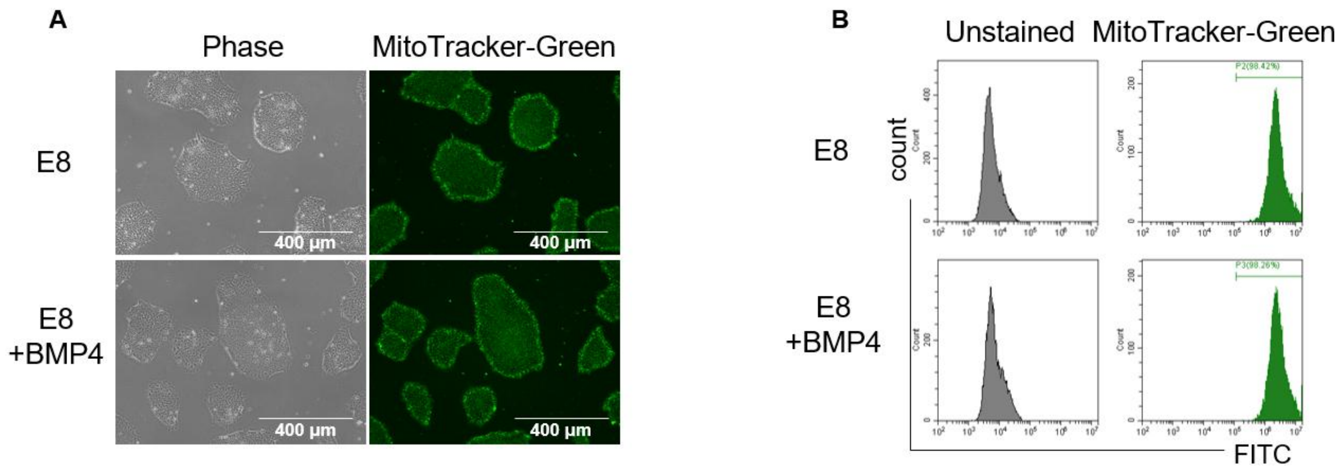


**Supplementary Figure 3.**

A-B. mitochondrial-mass analysis by mitotracker-green. The MitoTracker Green staining pattern was observed by fluorescence microscopy (A) and quantified by flow cytometry (FACS) (B).

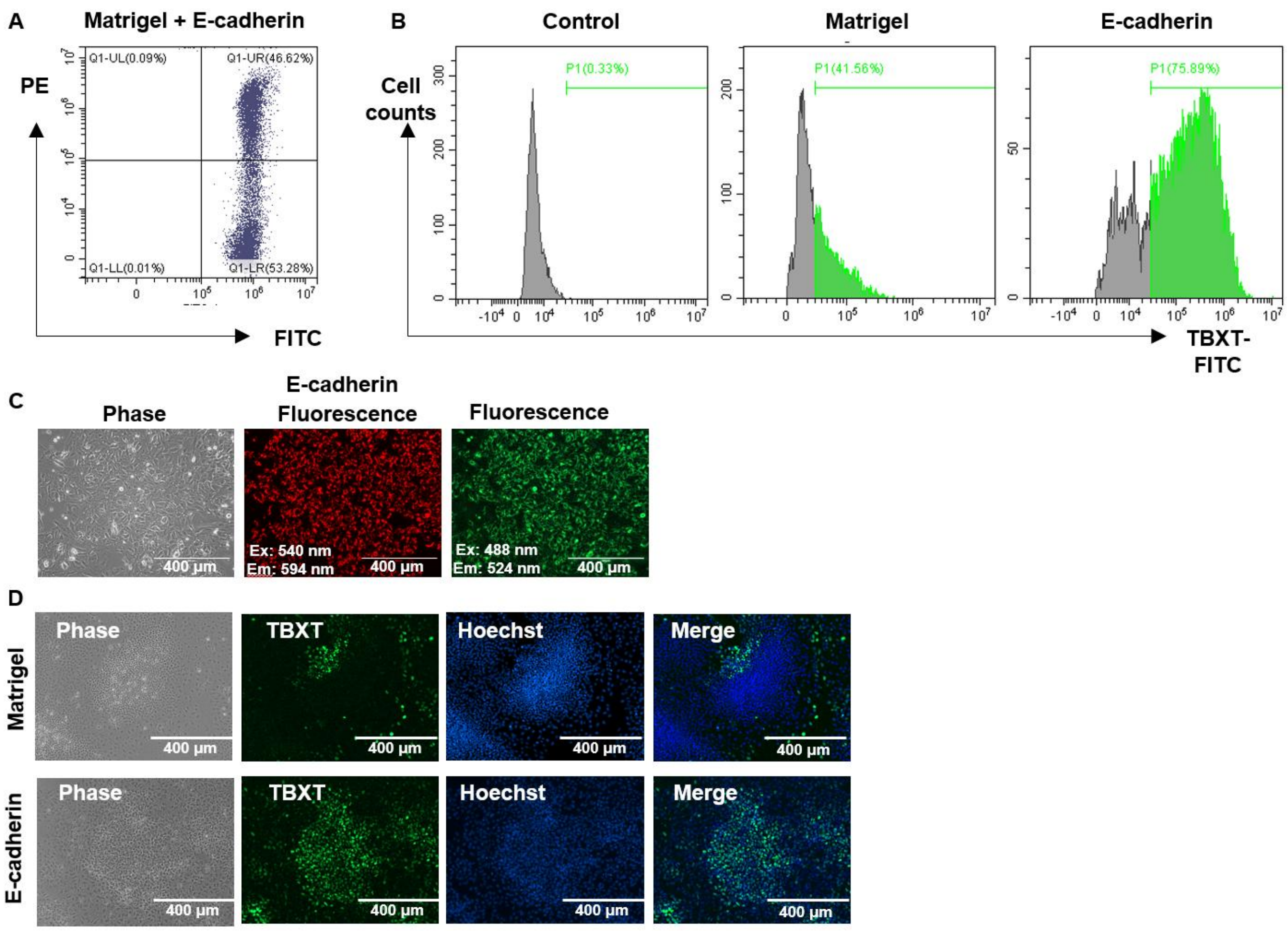


**Supplementary Figure 4.**

A. FACS analysis of JC-1 stained cells on Matrigel + E-cadherin coated surface.

B. FACS analysis showed more TBXT positive cells differentiated on E-cadherin coated surface than Matrigel coated surface.

C. hPSC H9 colonies were cultured on E-cadherin coated surface for two days and stained with JC-1.

D. Cell adhesion-promoting factors affected mesoderm differentiation induced by BMP4. hPSC H9 cells were cultured for 2 days on surfaces coated with either Matrigel or E-cadherin, prior to a 2-day induction with BMP4. Differentiation toward mesoderm was assessed by immunostaining for the marker TBXT.

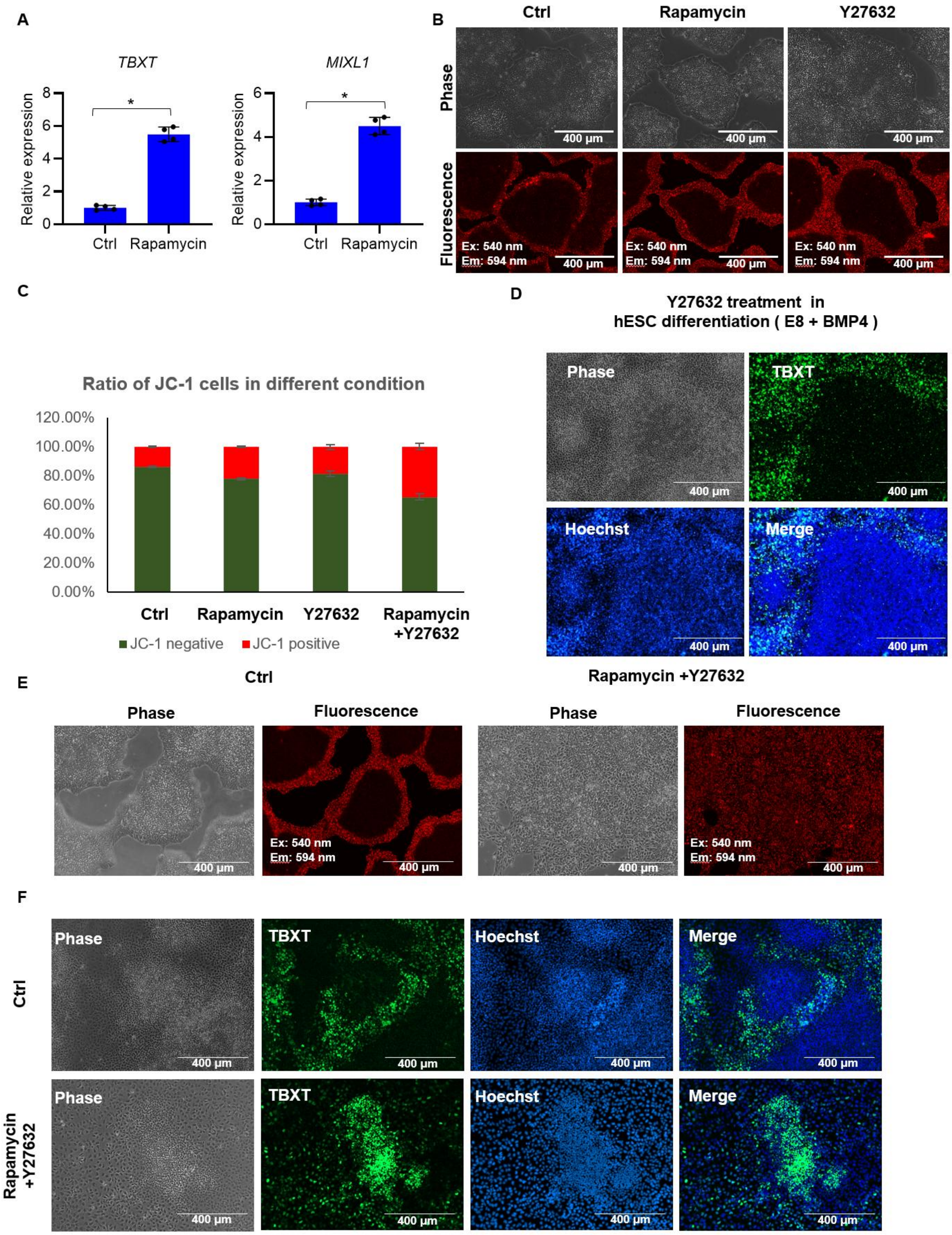


**Supplementary Figure 5.**

A. mTOR inhibition promotes mesoderm differentiation. Gene expression levels were

normalized to the control (Ctrl) group.

B-C. mTOR inhibition could not reverse the JC-1 stained pattern when the pattern had been formed. hESCs were passaged on matrigel coated surface, rapamycin was added after 24h removed Y27632, JC-1 stained pattern (B) was analyzed after 24h Rapamycin treatment, and also analyzed by FACS (n=3, * P<0.05)(C).

D. Y27632 didn’t’ affect BMP4 induced cell differentiation patterning. Immunostaining of TBXT on Day 4, following BMP4 induction with Y27632 on Day 2.

E. JC-1 patterns under mTOR and ROCK inhibition in hPSC H9 cells. hPSC H9 cells were passaged with 10 μM Y-27632 onto Matrigel-coated surfaces in E8 medium. Cells were then treated with either 100 nM Rapamycin (mTOR inhibitor) and 10 μM Y-27632 (ROCK inhibitor) on day 1 and day 2. JC-1 staining and microscopy observation were performed on day 3.

F. TBXT immunostaining in BMP4-induced hPSC H9 cells. hPSC H9 cells were treated with or without Rapamycin and Y-27632 for 24 hours prior to BMP4 induction on Day 2. Cells were then fixed and immunostained for the mesoderm marker TBXT on Day 4.

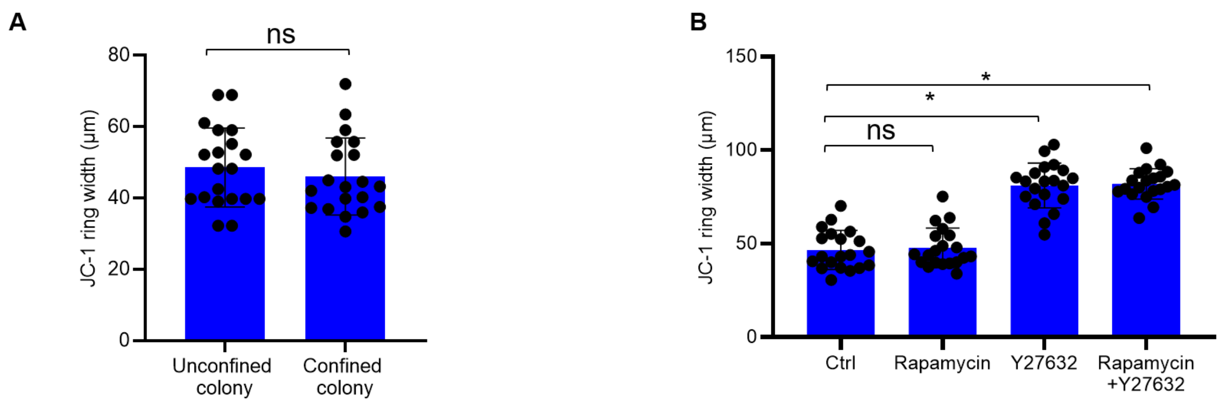


**Supplementary Figure 6.**

A. Image J analysis of JC-1 positive ring of hPSC colonies in confined culture. (n = 20 data points on the image; ns, not significant; * P<0.05).

B. Image J analysis of JC-1 positive ring of hPSC colonies treated by Rapamycin and Y27632 in confined condition. (n = 20 data points on the image; ns, not significant; * P<0.05).

**Supplementary table 1.** Primers used for real time PCR.

| Gene | Primer | Sequence |
|---|---|---|
| *GAPDH* | GAPDH-F | GTGGACCTGACCTGCCGTCT |
| | GAPDH-R | GGAGGAGTGGGTGTCGCTGT |
| *TBP* | TBP-F | CCACTCACAGACTCTCACAAC |
| | TPB-R | CTGCGGTACAATCCCAGAACT |
| *TBXT* | TBXT-F | ACCCAGTTCATAGCGGTGAC |
| | TBXT-R | CCATTGGGAGTACCCAGGTT |
| *MIXL1* | MIXL1-F | GGTACCCCGACATCCACTT |
| | MIXL1R | CGCCTGTTCTGGAACCATAC |
| *NANOG* | NANOG-F | CAGAAGGCCTCAGCACCTAC |
| | NANOG-R | ATTGTTCCAGGTCTGGTTGC |
| *SOX2* | SOX2-F | TACCTCTTCCTCCCACTCCA |
| | SOX2-R | GGTAGTGCTGGGACATGTGA |
| *POU5F1* | POU5F1-F | CAGTGCCCGAAACCCACAC |
| | POU5F1-R | GGAGACCCAGCAGCCTCAAA |
| *NOG* | Noggin-F | CCATGCCGAGCGAGATCAAA |
| | Noggin-R | TCGGAAATGATGGGGTACTGG |
| *SOX17* | SOX17-F | CGCACGGAATTTGAACAGTA |
| | SOX17-R | GGATCAGGGACCTGTCACAC |
